# Feature Selection and Network‐Driven Analyses to Unveil Common RNA Signatures in Colon and Pancreatic KRAS‐Mutant Cancers

**DOI:** 10.1002/cam4.70468

**Published:** 2025-02-27

**Authors:** Katia Pane, Mario Zanfardino, Anna Maria Grimaldi, Ilaria Leone, Silvia Nuzzo, Marco Salvatore, Monica Franzese

**Affiliations:** ^1^ Bioinformatics And Biostatistics Laboratory IRCCS SYNLAB SDN Naples Italy

**Keywords:** colon cancer, feature selection, KRAS mutation, network analysis, pancreatic cancer, prognosis

## Abstract

**Background:**

Colon cancer and pancreatic ductal adenocarcinoma are among the most aggressive tumors for which therapeutic options are limited. Both cancers share common features, such as some KRAS pathogenic variants and common epidemiology. The integration of multidimensional datasets by combining machine learning and bioinformatics approaches could provide deeper insights into the intricate KRAS‐related networks underlying cancer progression and unveil novel biomarkers and potential therapeutic targets. This study aimed to uncover colon and pancreatic cancers that shared transcriptional changes closely related to KRAS missense mutations.

**Methods:**

Feature Selection (FS) technique and Qiagen's Ingenuity Pathway Analysis (IPA) were used to combine DNA‐Seq and RNA‐Seq data from mutant and wild‐type (WT) KRAS colon and pancreatic tumor samples.

**Results:**

From the FS, we prioritized 70 genes (54 protein‐coding genes and 16 ncRNA‐coding genes) that were able to discriminate between WT and mutated KRAS patients. These genes were involved in KRAS signaling and other related processes, such as EMT signaling, glycolysis, apical junction, Wnt/beta‐catenin signaling, and IL‐2/STAT5 signaling. Using IPA, we identified a top‐scoring network of 19 upregulated genes in both tumor types stratified into mutant KRAS and WT KRAS samples. For a set of genes, qRT–PCR performed on colon and pancreatic representative cancer cell lines showed concordant expression trends when comparing colon‐dominant KRAS mutants versus WT KRAS and dominant pancreatic KRAS mutants versus WT KRAS, as expected according to in silico analyses.

**Conclusions:**

Our findings may provide insight into the common transcriptional signatures potentially underlying colon and pancreatic KRAS‐mutant cancers. However, further studies are needed to elucidate the diagnostic and prognostic value of targets identified as common features in our study.

## Graphical abstract

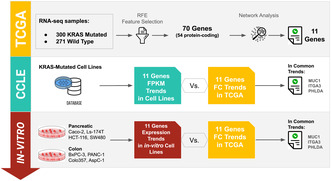

**Abbreviations**
anti‐EGFR: anti‐epidermal growth factor receptor
CGB8: chorionic gonadotropin beta subunit 8
DKK1: Dickkopf‐related protein 1
ECM1: extracellular matrix protein 1
ITGA3: integrin subunit alpha 3
MAPK: mitogen‐activated protein kinase
MUC1: Mucin 1
PADI1: peptidylarginine deiminase 1
PHLDA1: pleckstrin homology‐like domain, family A, member 1
PI3K‐AKT: phosphoinositide 3‐kinase, protein kinase B
PLEK2: pleckstrin 2
TGFA: transforming growth factor alpha
TGFBI: transforming growth factor beta induced
WNT7A: Wnt family member 7A

## 1 Introduction

Colorectal cancer (CRC) is the third most common human cancer and the second leading cause of cancer death worldwide [1, 2]. Moreover, pancreatic ductal adenocarcinoma (PDAC) constitutes approximately 85% pancreatic cancer and is one of the most aggressive and lethal cancers [2, 3]. Both cancer types are associated with the digestive system; they often exhibit late‐stage symptoms, high metastatic potential and limited therapeutic options [4]. Molecular studies have also revealed common colon and pancreatic genetic alterations that sustain tumor progression, such as Kirsten rat sarcoma viral oncogene homolog (KRAS) aberrations [5].

KRAS is most commonly mutated in pancreatic, colorectal, and lung adenocarcinomas [6, 7, 8, 9]. Moreover, KRAS somatic mutations are a hallmark of PDAC [9], making this gene the leading therapeutic target for intervention [10]. Somatic mutations in KRAS and other key genes also drive most CRC cases, whereas genomic instability (microsatellite instability, DNA repair defects, and chromosomal instability) mainly occurs in rare familial or hereditary CRC cases [11, 12, 13].

Several studies on the RAS oncogene family have shown that hotspot mutations occur differentially across cancers [7]. Three KRAS‐dominant missense mutations, G12, G13, and Q61, have implications for both colorectal and pancreatic cancers. All G12, G13, and Q61 mutants are gain‐of‐function, leading to constitutively active KRAS protein and dysregulated downstream signaling; for example, the mitogen‐activated protein kinase MAPK and PI3K‐AKT signaling cascades of KRAS mutants in G12D and G12V are present in 60%–70% of pancreatic ductal adenocarcinomas (PDAC) and 20%–30% of colorectal cancers (CRC) [7, 8, 14]. In CRC and PDAC, two common and dominant KRAS variants, G12D, are associated with reduced overall survival and poor prognosis, respectively [6, 15]. In advanced CRC, the KRAS codon G12 activating mutation coincides with multiple driver gene mutations to confer anti‐EGFR resistance [15]. Other potential commonalities between these two cancer types, as well as the intricate gene networks regulated by KRAS mutations, have not been explored.

**FIGURE 1:**
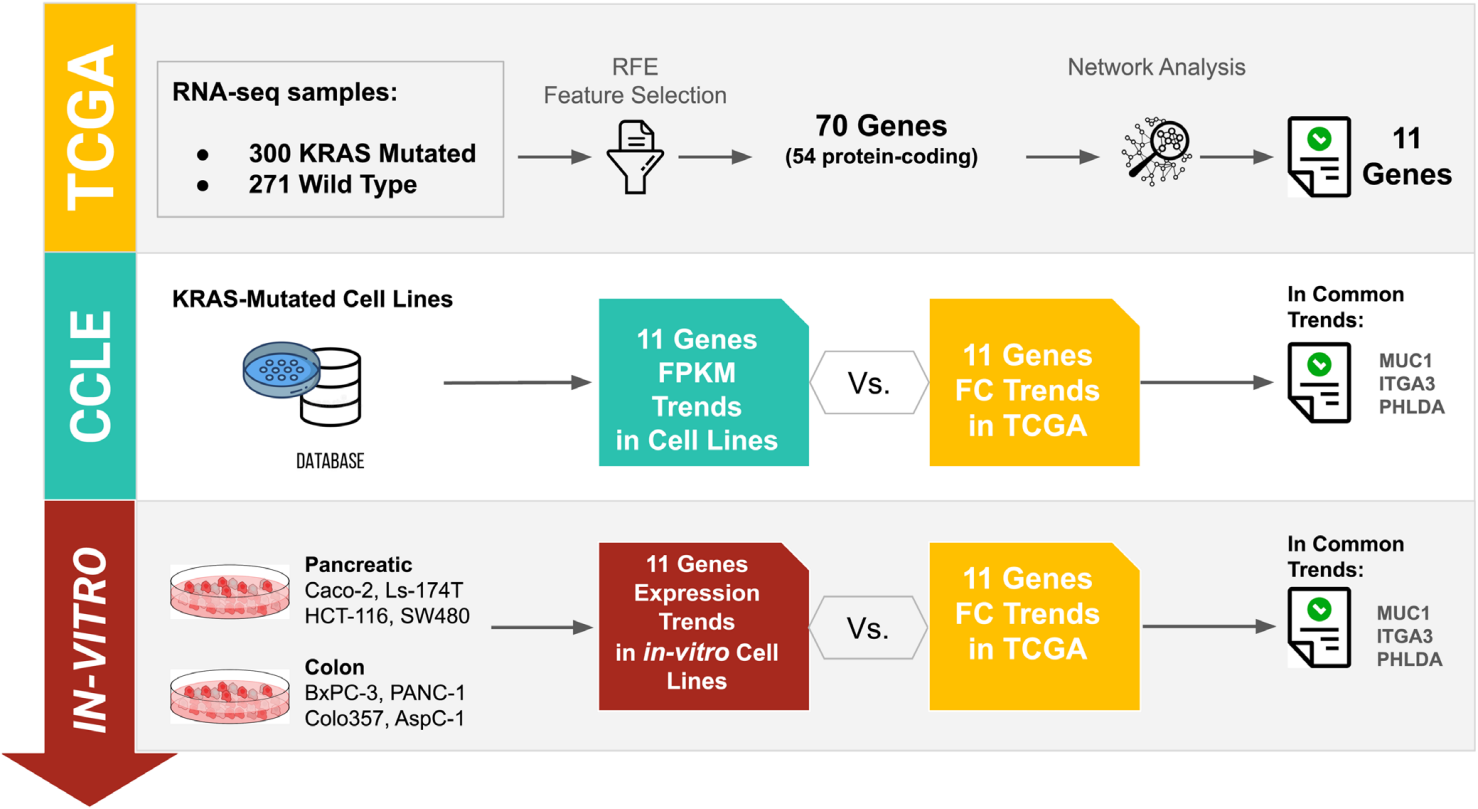
Methodological workflow. Our workflow involved the use of three successive steps: (1) The selection through Feature Selection technique (RFE: Recursive Feature Elimination) of genes capable of discriminating mutated KRAS samples from wild‐type samples. This first step was performed using data from TCGA (The Cancer Genome Atlas). (2) The validation of 11 selected genes using the CCLE (Cancer Cell Line Encyclopedia) cellular database. Finally, (3) validation on in vitro cell lines.

In modern medicine, machine learning (ML) approaches are receiving increased interest for precision medicine in treating CRC and PDAC [16, 17]. In this study, we attempted to identify shared transcriptional features underlying colon and pancreatic KRAS‐mutant cancers via an integrated ML‐based feature selection (FS) technique and network analysis framework (Figure 1). First, we analyzed TCGA DNA‐Seq data for subgrouping colon and pancreatic primary tumors bearing KRAS somatic mutations (mutKRAS) and WT KRAS (wtKRAS) via Qiagen's Ingenuity Pathway Analysis (IPA) software. Then, we integrated the transcriptome data of both tumor types with an FS method based on the ML technique. We prioritized 70 features (54 protein‐coding and 16 ncRNA) common to both cancers. Pathways and network analysis revealed that most of the identified genes were enriched in KRAS‐related pathways and involved in cancer disease, the cell cycle, cellular movement, and developmental disorders. We focused on 11 hub genes in the top‐scoring protein–protein network 1 and performed a through characterization of their expression changes by Cancer Cell Line Encyclopedia (CCLE). Then, we performed in vitro experiments on cells representative of the most common colon and pancreatic KRAS‐mutated subtypes as follow: pancreatic PANC‐1 (*KRAS* p.G12D), BxPC‐3 (*KRAS*‐WT), AsPC‐1 (*KRAS* p.G12D), and COLO 357 (*KRAS* p.G12D) cell lines and colorectal Caco2 (KRAS‐WT), HCT116 (*KRAS* p.G13D), SW480 (*KRAS* p.G12V), and Ls174T (*KRAS* p.G12D) cell lines. A total of 4 out of the 11 candidate genes showed a perfect concordant trend in both cancer representative cell lines. This study offers a perspective on the master activator *KRAS* oncogene and shared genetic and signaling pathways altered in these two aggressive cancer types, shedding light on potential actionable genes. Moreover, our findings provide an overview of the expression trends of relevant cancer‐related genes (in the tissues and cell lines associated with the analyzed tumors), considering the distinct mutational status of *KRAS*.

## 2 Methods

### 2.1 KRAS Somatic Mutation in TCGA, Data Integration, and Preprocessing

We used TCGA DNA‐Seq data for colorectal and pancreatic cancer cohorts generated with IPA Land Explorer QIAGEN bioinformatic software [18] and queried in July 2023 (version 94302991, date May 27, 2023). We assessed the DNA‐Seq somatic mutation distribution across the TCGA cohorts for the KRAS gene. We analyzed the TCGA‐COAD and TCGA‐PAAD cohorts, sample IDs, sample types, and DNA‐Seq somatic mutation KRAS statuses. In the TCGA‐COAD cohort, there were *N* = 171 “MUT” KRAS missense mutations and *N* = 228 “WT” wild‐type (WT) KRAS primary tumor; other missing samples. In the TCGA‐PAAD cohort, there were *N* = 137 “MUT” KRAS missense mutations and *N* = 43 “WT” wild‐type KRAS primary tumors. We stratified the RNA‐Seq data according to the KRAS DNA‐Seq status (wild‐type [WT] or mutant [MUT]).

### 2.2 Gene Expression Data

RNA‐Seq gene quantification (FPKM HT‐seq‐normalized reads) and clinical information for the TCGA COAD and PAAD cohorts were downloaded via the R package TCGABiolinks v2.14.1 [19]. The RNA‐Seq data for the primary tumors included *N* = 271 “WT” samples overall and *N* = 300 “MUT” samples overall (missing seven samples in TCGA‐PAAD and one sample in TCGA‐COAD). For all the data, we performed global data normalization via the upper quartile method (UQUA).

### 2.3 Machine Learning Analysis

Using the default Caret package, our dataset was split into training and test sets for the FS step, preserving the proportion of cancer types in the data distribution (the same percentage of cancer types as in the full dataset, training dataset, and test dataset). Then, we performed FS using the recursive feature elimination (RFE) method (caret v6.0‐88) and tested different subsets of features (from a group of 20 to 100 genes, step = 10), which resulted in the selection of 70 genes (of whom 54 protein‐coding biotypes) with the best accuracy. To perform internal classification in FS, we used the random forest‐based model. In the training phase, we applied *k*‐fold cross‐validation (*k* = 10).

### 2.4 Pathways and Regulatory Network Analyses

We assessed the most enriched terms associated with the 70 common signatures by querying g:Profiler version e111_eg58_p18_30541362, database updated on January 25, 2024 [20]. The associations between KRAS mutation‐related genes and their regulatory networks were more insight investigated by using IPA QIAGEN bioinformatics software. We used a bubble chart to show the IPA canonical pathway names versus pathway categories with a *p* value < 0.05 according to the right‐tailed Fisher's exact test, with bubbles colored associated to the *z* score by gene expression changes (log_2_FC, contrast mutant vs. WT KRAS tumor samples). Using the IPA Knowledgebase (genes only), we found six common networks with relationship scores ranging from 46 to 3. We deep biologically insight on the top scoring network, including 19 overlapping focus molecules of the 70 genes.

### 2.5 KRAS Somatic Mutation in CCLE and Data Analysis

We queried the Cancer Cell Line Encyclopedia (CCLE) dataset (PRJNA523380) with the latest CCLE consortium manually curated annotations. By searching for CCLE matching large intestine and pancreas ID, we found *N* = 100 cell lines including *N* = 57 colorectum, *N* = 41 pancreatic, and *N* = 2 control. This latter, being two fibroblast lineages (HS255T and HS675T), were removed. We joined the CCLE annotations for KRAS somatic mutations with the RNA‐Seq expression data for the 11 genes of interest for a total of 98 cell lines covering 15 KRAS distinct missense mutations. We kept the colorectal and pancreatic cancer cell lines harboring the six most common KRAS missense mutations at Hotspots 12, 13, and 61 [7] leading to KRAS protein changes such as “G12D,” “G12V,” “G13D,” “G12R,” “Q61H,” and “G12C” present in the CCLE cell lines for colon and pancreatic cancers as well as in the TCGA‐COAD and TCGA‐PAAD primary tumors (Figure S1), by ggvenn v. 0.1.10. Expression profiles of the 11 genes were plotted as (log_2_(FPKM + 1)) clustered by row dendrogram generated by ComplexHeatmap_v. 2.2.0 [21].

### 2.6 Cell Lines Culture and qRT–PCR Experiments

Human colon cancer cell lines (Caco‐2, Ls‐147T, HCT‐116, and SW480) and human pancreatic cancer cell lines (BxPC‐3, PANC‐1, Colo 357, and AspC‐1) were cultured according to the manufacturer's recommendations. Briefly, Caco‐2 cells (ACC 169) were cultured in 80% MEM (with Earle's salts) supplemented with 20% fetal bovine serum (FBS) and 1× nonessential amino acids. SW480 cells (DMSZ #ACC‐313) were grown in 90% RPMI 1640 supplemented with 10% h.i. FBS. HCT116 cells (DMSZ #ACC 581) were cultured in 90% McCoy's 5A, 10% FBS, and 2 mM l‐glutamine. Ls‐174T cells were grown in 90% Eagle's minimum essential medium supplemented with 10% FBS. Regarding the pancreatic cell lines, BxPC‐3 (CRL‐1687), AsPC‐1 (CRL‐1682), and Colo 357 were cultured in 90% RPMI 1640 supplemented with 10% FBS. PANC‐1 cells (CRL‐1469) were grown in Dulbecco's modified Eagle's medium (DMEM) supplemented with fetal bovine serum (FBS) to a final concentration of 10%. Total RNA was isolated from cell cultures in triplicate using TRIzol reagent according to the manufacturer's instructions. The RNA quantity and quality were evaluated by a NanoPhotometer NP80 (IMPLEN, USA) based on the A260/280 and A260/230 ratios. cDNA was synthesized from three replicates per cell line using SuperScript IV VILO Master Mix (Invitrogen, USA). For cultured cells, the expression levels of *CGB8*, *DKK1*, *ECM1*, *MUC1*, *PADI1*, *PHLDA1*, *PLEK2*, *ITGA3*, *TGFA*, *TGFBI*, and *WNT7A* were determined via qRT–PCR using specific primers (Table S1). All the reactions were carried out with iQ SYBR Green Supermix (Bio‐Rad, USA) on a CFX384 real‐time PCR system (Bio‐Rad) using the following conditions: 95°C for 15 min; 40 cycles of 94°C for 15 s; 55°C for 30 s and 72°C for 30 s. The maximum cycle threshold (Ct) was set at 40. β‐ACT was used as a housekeeping control gene. All experiments were carried out in triplicate for each data point, and data analysis was conducted using CFX Maestro Software (Bio‐Rad). The data are expressed as relative expression levels (compared with those of the KRAS‐WT cell line) and were calculated using the $2^{-\Delta \Delta C_{t}}$ method. To assess the statistical analysis of the qRT–PCR data, we first performed Shapiro–Wilk tests to confirm the normality of the data, which were subsequently analyzed via one‐way ANOVA with Tukey's post hoc test via GraphPad Prism 9.0 software. A *p* value of < 0.05 was considered to indicate statistical significance.

## 3 Results

### 3.1 KRAS Somatic Mutation Distribution in the TCGA Cohort

**FIGURE 2:**
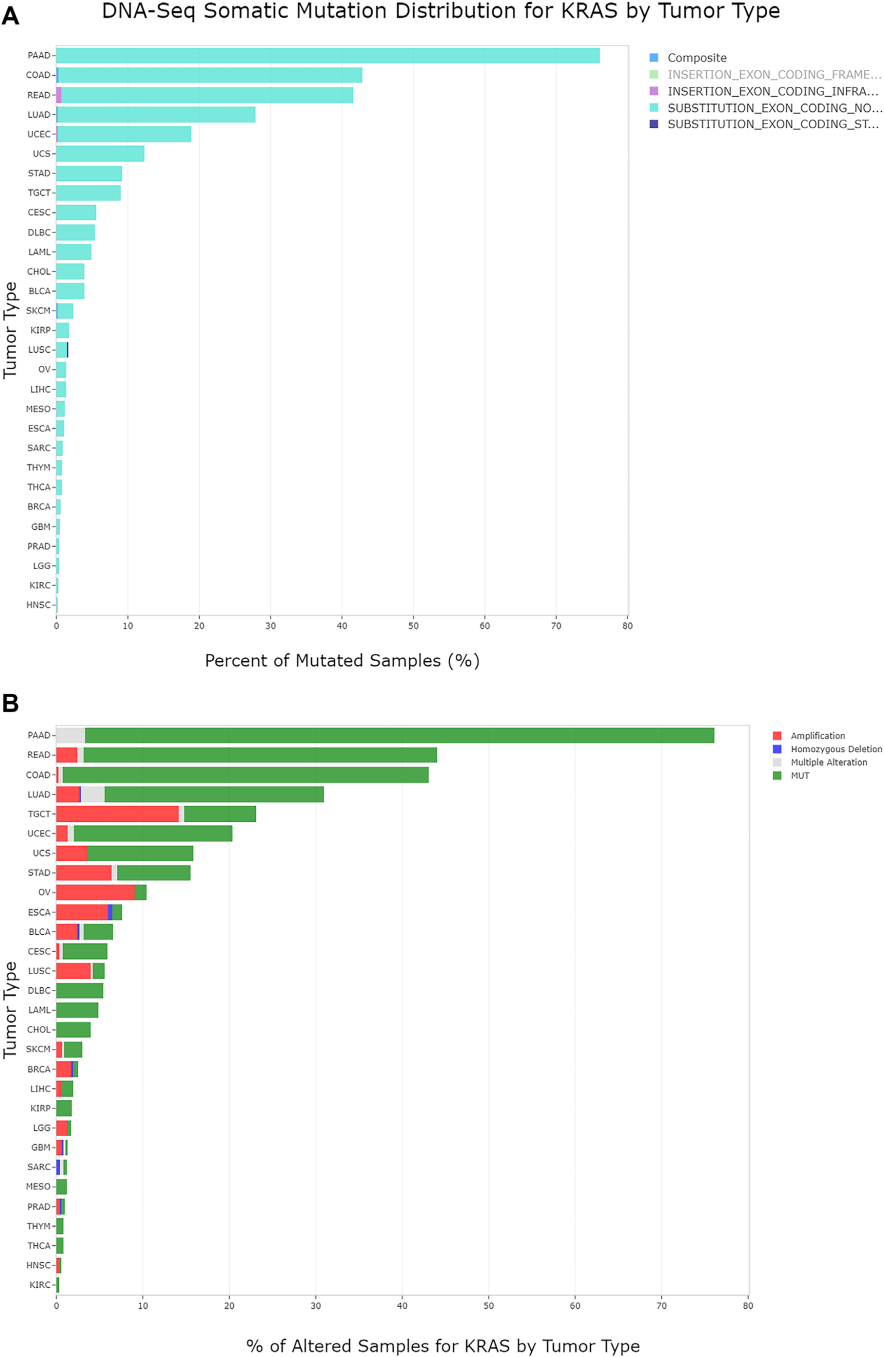
Bar plot of KRAS gene alterations in all TCGA datasets stratified by tissue type. (A) The highest percentage of mutated samples were in the pancreatic, colon, and rectal cancer dataset. (B) The most common missense mutations were “Substitution_exon_coding_non_synonymous.”

We used IPA Land Explorer as a prior knowledge base to integrate somatic mutation data with other omics data, such as gene expression and clinical information. In all TCGA cohorts, exploratory analysis of the DNA‐Seq somatic mutation distribution for KRAS according to tumor type confirmed the occurrence of this mutation in the literature [15, 22]. Indeed, the highest percentage of *KRAS*‐mutated samples were from pancreatic, colon and rectal cancers (Figure 2). Moreover, the most common KRAS genetic alterations in all TCGA cohorts were missense mutations, named “Substitution_exon_coding_non_synonymous” (Figure 2), which had the highest frequencies in PDAC and COAD tumors. The IPA was used to analyze patient primary tumors based on *KRAS* somatic mutation status. Therefore, we sub‐grouped pancreatic and colon primary tumors into “MUT” having “somatic *KRAS* missense mutation” and “WT” samples.

### 3.2 Feature Selection

The FS process using RFE resulted in the identification of an optimal subset of 70 genes, 54 of which were associated with the protein‐coding biotype. This selection was based on achieving the best performance in terms of accuracy (0.74) during testing with different subsets of features ranging from 20 to 100 genes (Table S2). For each feature, we also obtained a measure (weight) of how each variable contributes to discriminating the outcome (mutated vs. WT) used to train a random forest model in the FS process. The feature weight is linearly proportional to the model feature importance.

### 3.3 Pathways and Network Analysis

**FIGURE 3:**
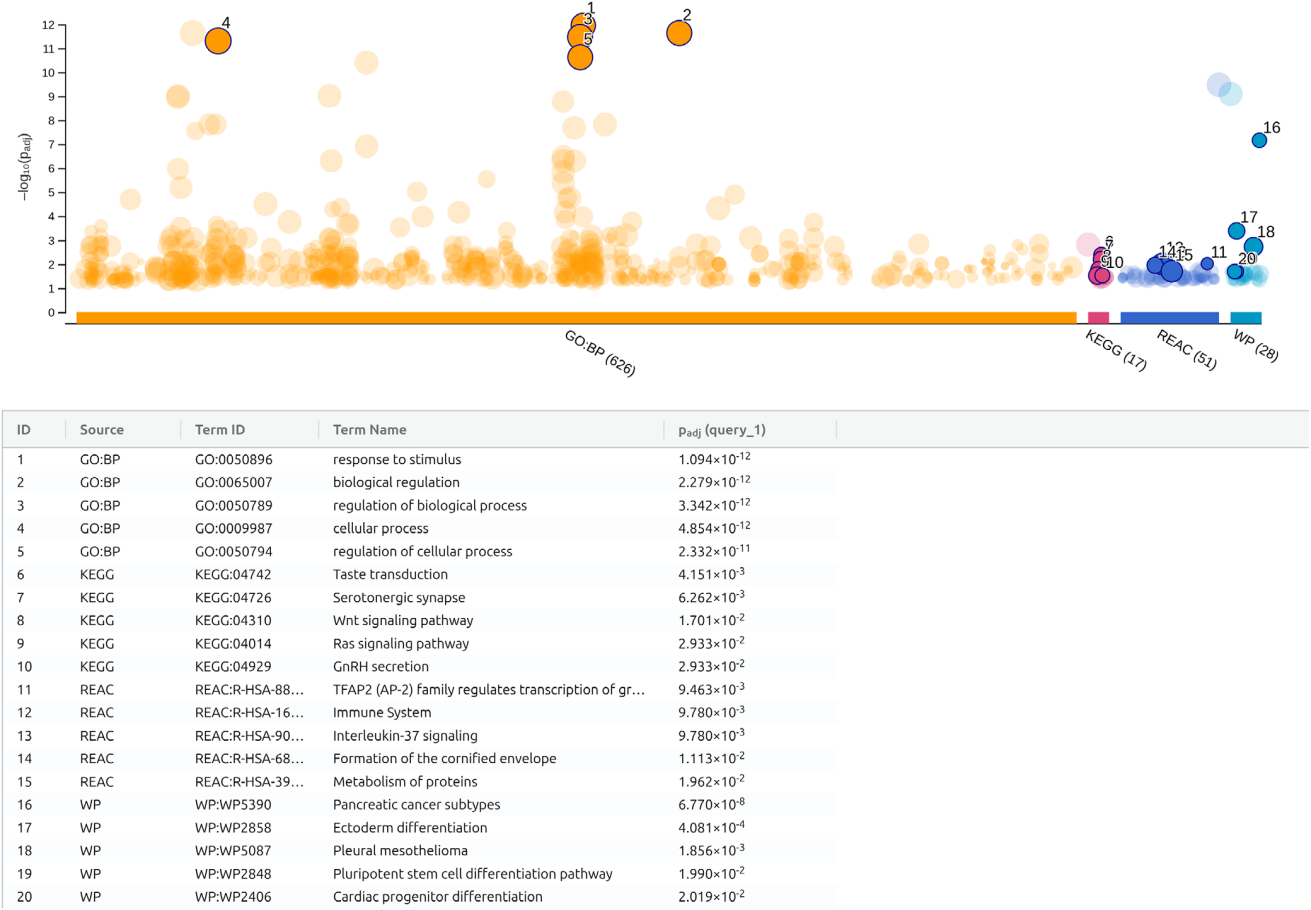
g:Profiler analysis. Top 20 pathways resulting from g:Profiler tool using GO:BP (Gene Ontology:Biological Process), KEGG (), Reactome, and WikiPathways database.

**FIGURE 4:**
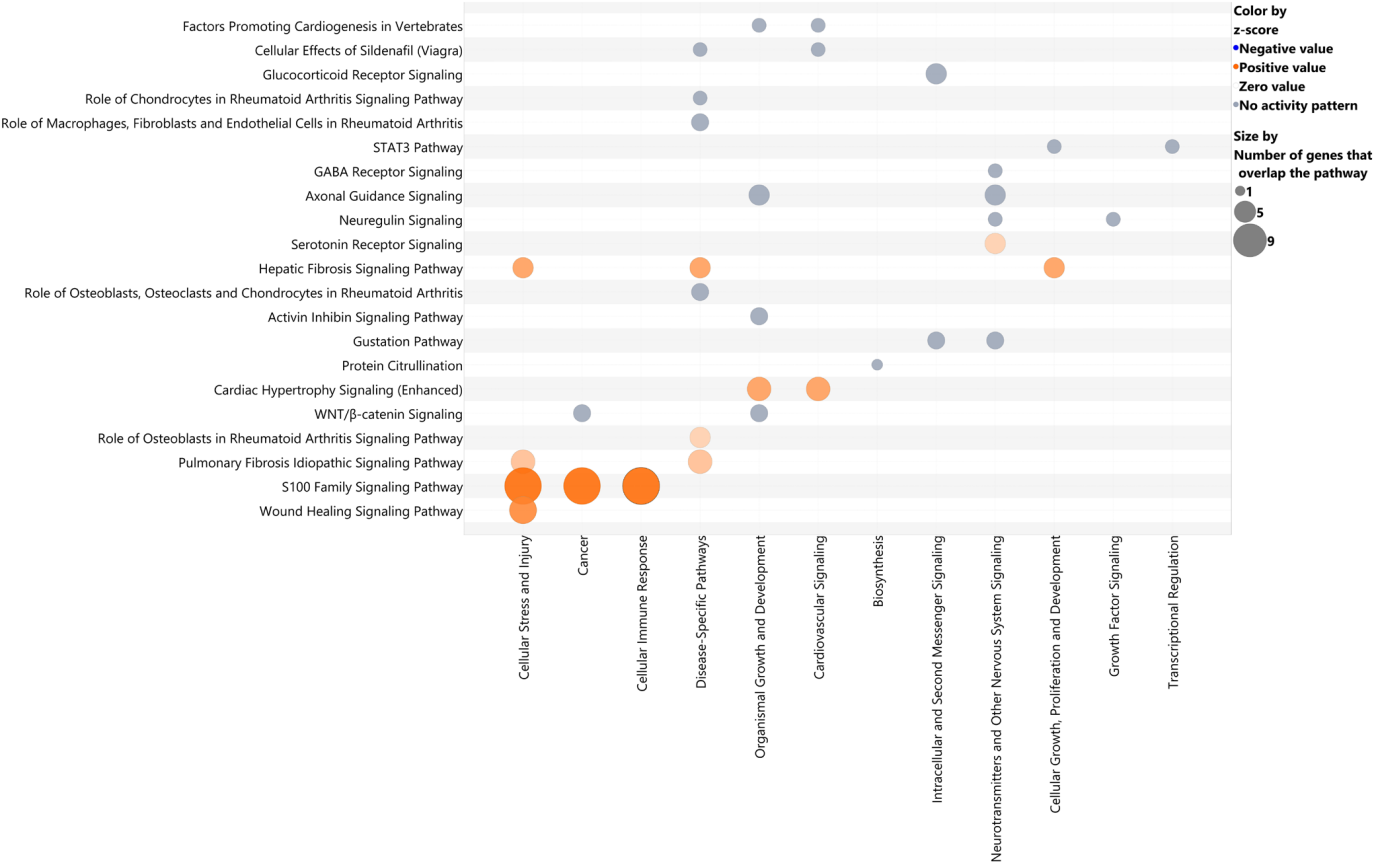
Functional enrichment analyses of the 70 genes. Bubble chart of the IPA canonical pathways name versus pathway category with a *p* value < 0.05 right‐tailed Fisher's exact test. Gene expression data derived from log_2_FC values (mutant KRAS vs. wild‐type KRAS tumor samples). Bubbles are colored according to *z* score, and the largest bubbles have the highest number of overlapping genes within that pathway.

**FIGURE 5:**
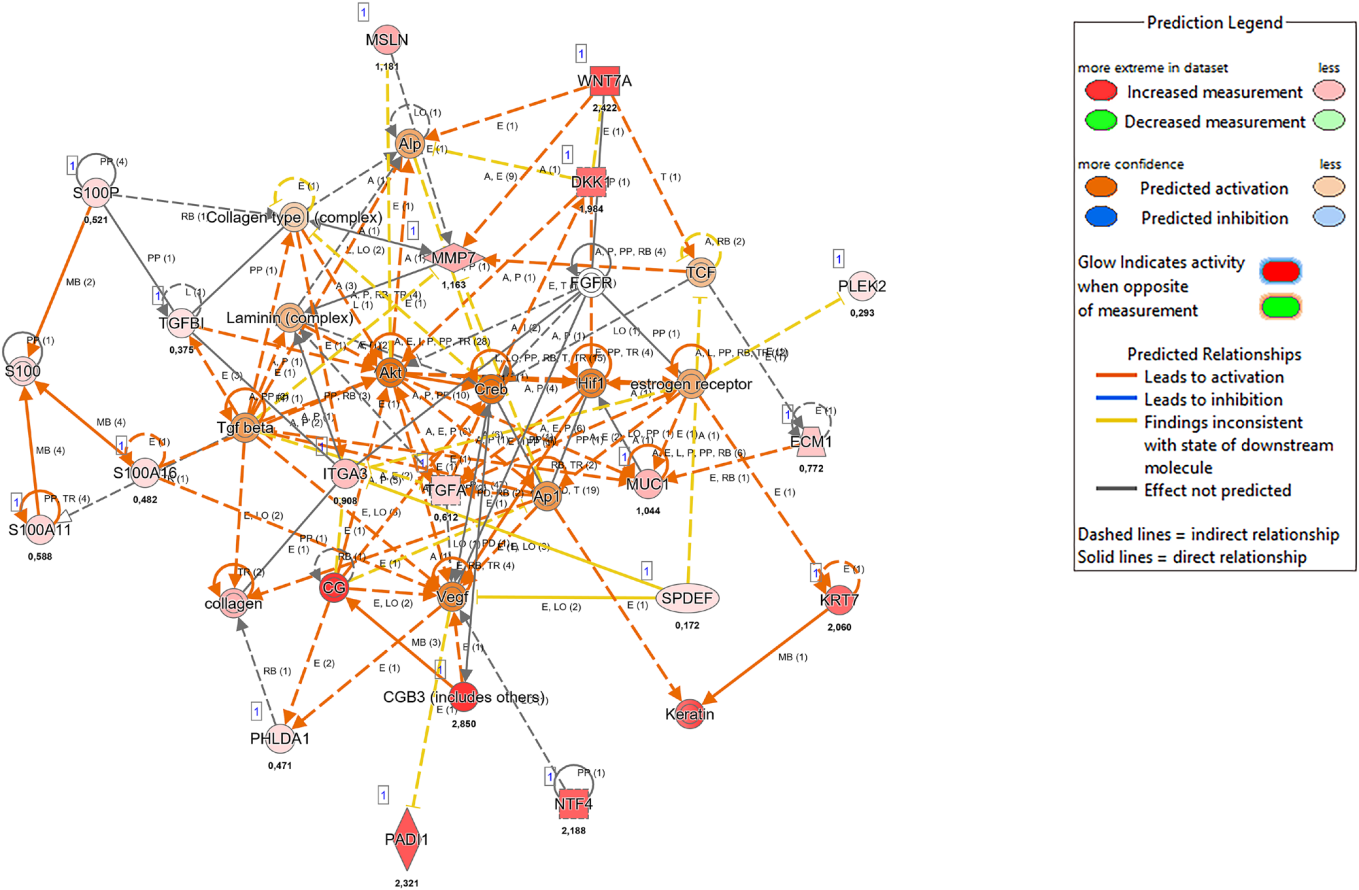
IPA top‐scoring network. Network 1 focused on 19 out of 70 input molecules. Prediction activity generated by expression changes and IPA Knowledge Base software.

We performed g:Profiler enrichment analysis query distinct databases for the 70 common features. The most enriched terms by Reactome, Wikipathways, KEGG and Gene Ontology for biological processes highlighted the *Wnt Signaling Pathway*, the *RAS Signaling pathways*, and top scoring term *Pancreatic cancer subtypes* present in Wikipathways (Figure 3 and Table S3). Then to deep biologically insight, we leveraged the IPA knowledgebase to assess the canonical pathways and putative regulatory network associated with the 70 common features. As shown in Figure 4, the most enriched biological processes involved tumorigenic and immunomodulatory pathways underlying CRC and PDAC in the early phase confirming the previous tool findings. IPA enriched terms fall within the S100 protein family signaling pathway and the Wnt/beta‐catenin signaling pathway, which are evolutionarily conserved pathways with major roles in cellular homeostasis and differentiation, and other key pro‐growth pathways consistent with the loss of the fine‐tuned KRAS molecular switch. We discovered six networks according to the Ingenuity Knowledge Base (Genes Only) direct relationship (Figure 5). The top‐scoring network included 19 candidates (protein‐coding genes) as focused molecules involved in cancer disease and the cell cycle, cellular movement, and developmental disorders (Table S4). Indeed, the Wnt family member 7A and a member of the Dickkopf family act in direct and indirect manners as negative regulators of *WNT* signaling and are widely studied for regulating differentiation, polarity, and migration; as such, they are also involved in cell transformation. DKK1, a protein upregulated in human serum, has been recently associated with pancreatic cancer [23, 24]. Integrin subunit member 3 (*ITGA3*), which acts as an adhesion molecule and is also involved in tumor biology, as well as the extracellular matrix protein 1 (*ECM1*), is associated with endochondral bone formation and angiogenesis. High levels of ECM1 exosomes have been identified in CRC patients with a higher risk of relapse [25]. Among cancer hallmarks, glucose metabolism is also altered; for example, in Network 1, the Mucin1 gene (*MUC1*) are overexpressed in several carcinomas and are related to the hypoxic response in pancreatic cancer cells [26]. Regarding the epithelial–mesenchymal transition, Network 1 also included transforming growth factor alpha (*TGFA*), which promotes epithelial development, proliferation, and differentiation through the epidermal growth factor receptor (EGFR) signaling pathway. Moreover, among the focused molecules are transforming growth factor Beta 1 (*TGFB1*), which is dysregulated in multiple cancers and in the basal type of PDAC, conferring poor outcomes [27, 28]. Recently, specific inhibitors have proven effective in non‐small cell lung cancer (NSCLC) [29]. The pleckstrin 2 gene (*PLEK2*) encodes a membrane‐bound phosphatidylinositol generated by phosphatidylinositol 3‐kinase; this gene is a canonical downstream effector of *KRAS* activation, and its overexpression has been associated with colorectal cancer [30]. Finally, the pleckstrin homology‐like domain in family A (*PHLDA1*) has implications for intestinal tumorigenesis, as demonstrated by siRNA‐mediated suppression of PHLDA1 in colon cancer cells [31]. The Molecular Signature Hallmark database, as an external database, confirmed that the Network 1 genes *WNT7A*, *DKK1*, *ITGA3*, *ECM1*, *MUC1*, *TGFA*, *PHLDA1*, *TGFBI*, and *PLEK2* were enriched in *KRAS signaling* and other related terms, such as *EMT signaling*, *glycolysis*, *apical junction*, *Wnt/beta‐catenin signaling*, and *IL‐2/STAT5 signaling* (data not shown). For a thorough characterization of these promising common features in distinct KRAS colorectal and pancreatic cell mutation context, we assessed the expression levels of these nine genes and a member of the chorionic gonadotropin protein, *CGB8*, and the enzyme peptidyl arginine deiminase 1 (*PADI1*), which are involved in Protein Citrullination, because of their high expression changes and recent implications in many cancers [32, 33, 34].

### 3.4 KRAS Mutant and Wild Type in Colorectal/Pancreatic Cells From CCLE

**FIGURE 6:**
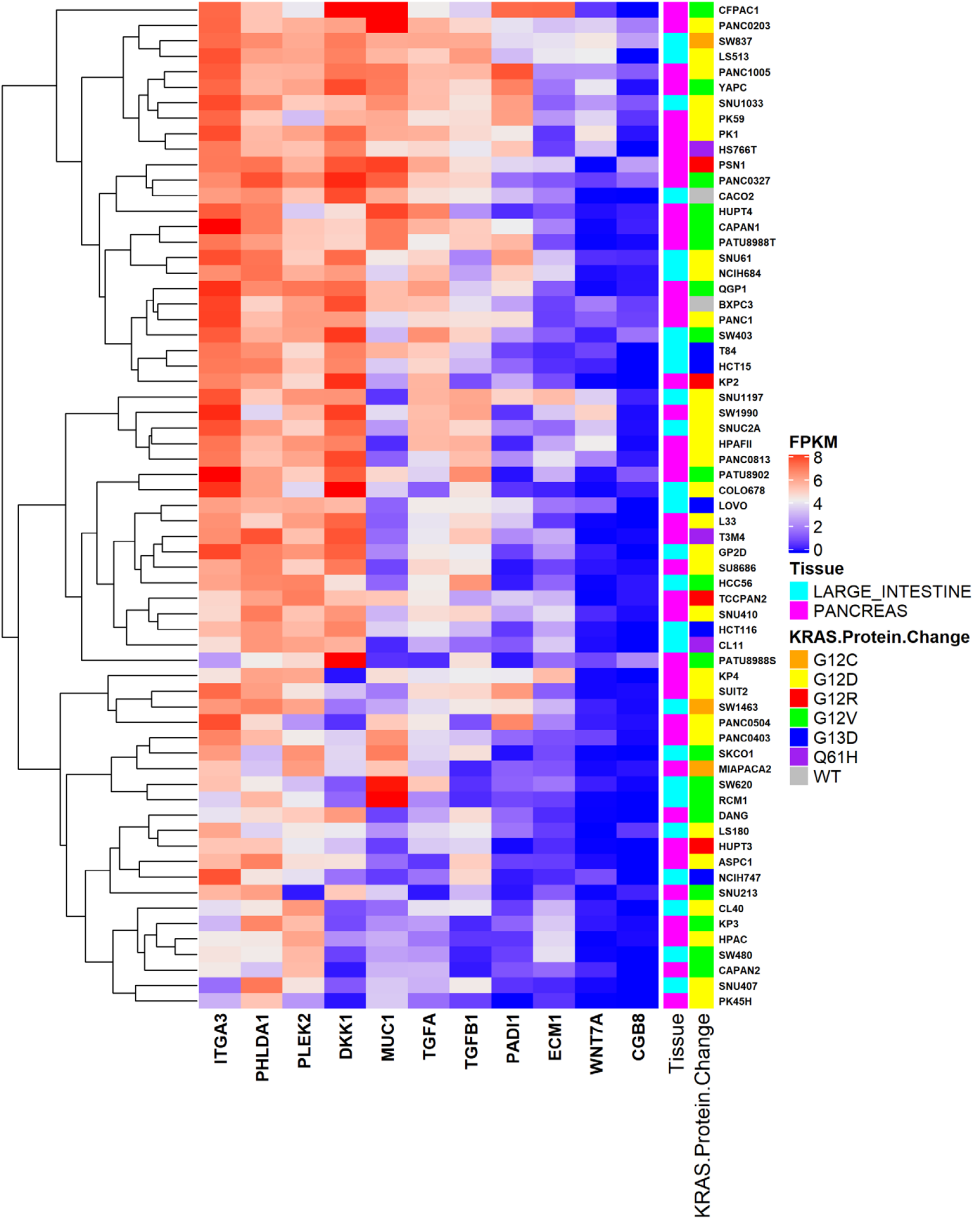
CCLE characterization of colorectal and pancreatic cell lines. Heatmap showed the expression profiles (log_2_(FPKM + 1)) of 11 common features prioritized by network analysis, across colorectal and pancreatic cancer, and parental cell lines from CCLE.

We explored in silico the transcriptomics profiles for the 11 selected genes by RNA‐Seq data from the CCLE joining for each colorectal and pancreatic tumor type the most common KRAS missense mutations (hotspot 12, 13, 61) [7]. Heatmap showed the expression profiles of the 11 genes (column) across 65 CCLE cells (row) (Figure 6). Additional annotations reported *N* = 25 and *N* = 38 large intestine and pancreas tissue, respectively including KRAS protein changes as follow 2 KRAS‐WT (in gray, CACO2 and BxCP3 colorectal and pancreatic cell line, respectively), G12D (in yellow, 46%), G12V (in green, 30%), G13D (in blue, ~8%), and G12R (in red, ~6%), G12C (in orange, ~4.7%), and Q61H (in purple, ~4.7%). When plotted against the colorectal and pancreatic cell lines from CCLE, the left subset of genes was characterized by relatively high expression for both cancer cell line types (highlighted in light red) while the subset of genes on the right side showed relatively low expression (highlighted in light blue). To further evaluate the expression trend of these common signatures we carried out in vitro validation in KRAS‐mutant and KRAS‐WT colorectal and pancreatic cell lines.

### 3.5 In Vitro Validation

**FIGURE 7:**
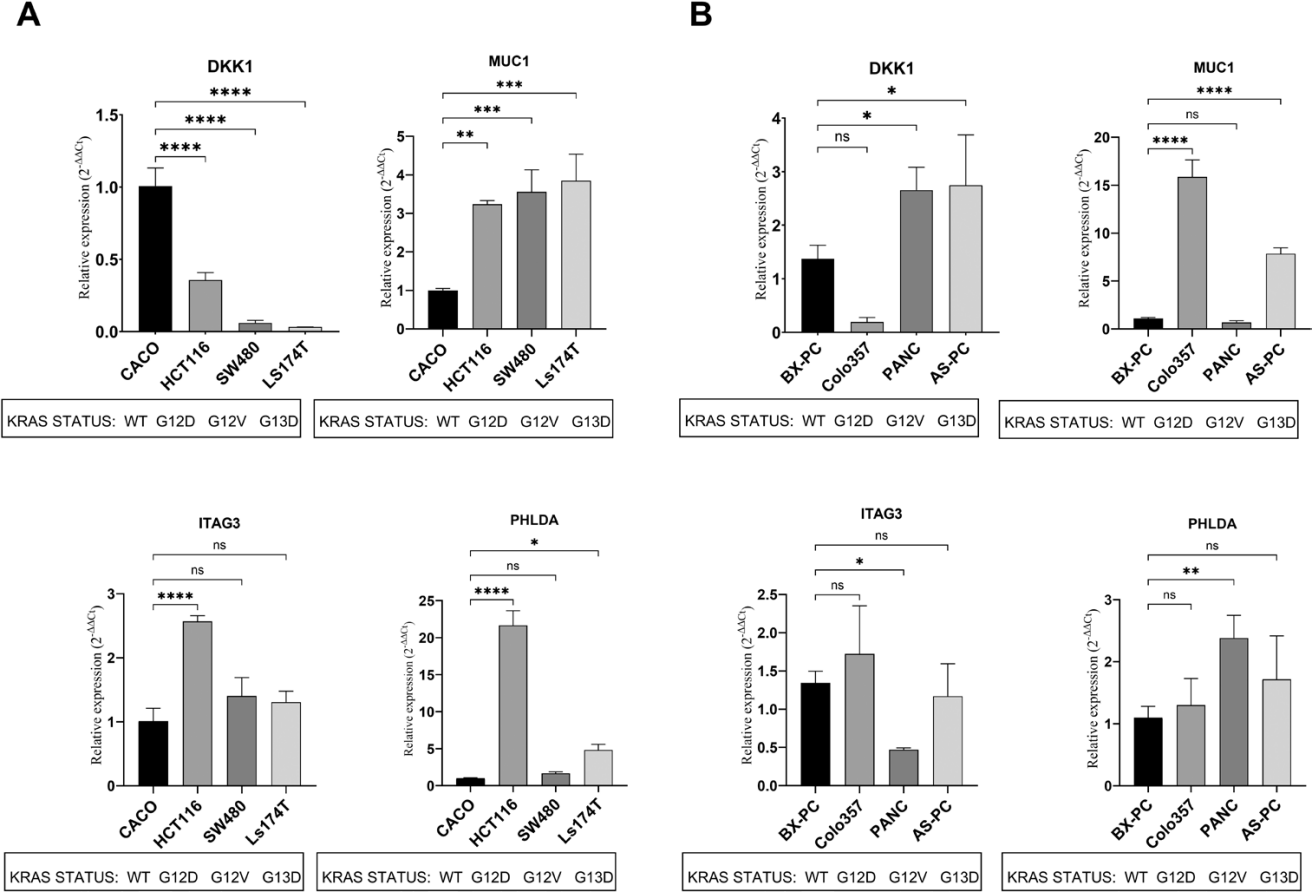
qRT–PCR analysis of the mRNA levels of *DKK1*, *MUC1*, *ITGA3*, and *PHLDA*. (A) Changes in the relative expression of the human pancreatic cancer cell lines BxPC‐3 (KRAS‐WT) and the mutant KRAS cancer cell lines PANC‐1, Colo 357, and AspC‐1. (B) Relative changes in expression in humancolon Caco‐2 (KRAS‐WT) and mutant KRAS cancer cell lines Ls‐147T, HCT‐116, and SW480. All the experiments were carried out in triplicate for each data point, and the results are expressed as the relative expression level calculated using the $2^{-\Delta \Delta C_{t}}$ method (compared with that of the *KRAS*‐WT cell line). A *p* value of < 0.05 was considered to indicate statistical significance (one‐way ANOVA with Tukey's post hoc test). *p value < 0.05, **p value < 0.01, ***p value < 0.001, ****p value < 0.0001.

We selected a panel of cancer cell lines representative of the most common colon and pancreatic subtypes according to their molecular phenotypes [35, 36, 37] (Table S5). qRT–PCR was used to confirm the identified network‐based gene signatures by determining the relative expression of 11 genes. *MUC1*, *ITGA3*, and *PHLDA1* were overexpressed in a statistically significant manner in at least one or more mutant *KRAS* cell lines compared to WT KRAS cells (Figure 7). This trend in expression agreed with the results obtained by in silico prediction. In vitro experiments confirmed the expected changes in the expression of most genes in the CRC‐MT and ‐*KRAS*‐WT cancer cell lines (Table 1). Indeed, all the genes were overexpressed in the CRC mutant compared to the *KRAS WT* cancer cell lines, except for *DKK1* and *ECM1*. However, while for *DKK1*, a decreasing trend was common to all three KRAS‐mutated cell lines, for *MUC1*, the mutated cell line Ls174T (p.G12D) showed a trend toward overexpression, albeit the difference was not statistically significant.

**TABLE 1:** Expression changes for the majority of genes in CRC mutant vs KRAS wild type cancer cell lines.

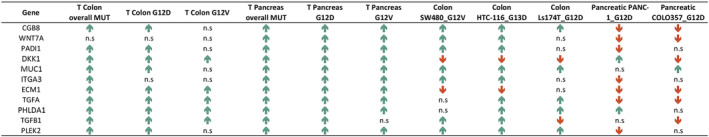

In contrast to those in CRC, the expression of selected network‐based genes in PDAC‐mutant KRAS cell lines exhibited greater heterogeneity, and the trend in expression was associated with each mutant form of *KRAS*. In addition to the three genes that are commonly overexpressed in the colon, *DKK1* was also significantly upregulated in two of the PDAC *KRA*S‐mutated cell lines (PANC‐1 and AsPC‐1). The remaining genes in the tested *KRAS*‐mutated cell lines appeared to be under expressed or poorly altered overall.

## 4 Discussion

*KRAS* signaling has historically been considered undruggable due to its intricate and multifaceted network of downstream effectors. The molecular heterogeneity of *KRAS* mutants poses challenges for identifying effective therapeutic targets and poorly defined *KRAS* epitopes remain [18, 38, 39, 40]. Targeting KRAS‐mutant carcinomas is also challenging due to the tissue‐specific pattern of KRAS codon mutations [39]. Furthermore, inhibitors of mutans KRAS have proven ineffective in clinical, emphasizing the importance of discovery drugs targeting downstream KRAS pathways. In CRC and PDAC, three hotspots for KRAS missense mutations (G12, G13, and Q61) lead to KRAS oncoproteins sustaining proliferative signaling, mainly through the *MAPK* and *PI3K‐AKT* signaling pathways [8]. Recently, clinical studies have proven effective at targeting oncogenic KRAS point mutations, such as the G12C mutation, but additional efforts are needed to determine the major genetic alterations affecting CRC and PDAC [4]. In particular, G12D (glycine 12 to aspartic acid) and G12V (glycine 12 to valine) are the two most common subtypes of CRC [8] and G12D in PDAC [6].

In our study, we attempted to identify common transcriptional signatures underlying *KRAS* mutations in WT CRC and PDAC. Our integrated analysis, using an ML‐based FS prioritized 70 features able to distinguish colon and pancreatic *KRAS*‐mutant versus WT cancers with an AUC equal to 0.74. This subset of genes was enriched in key cancerogenic pathways underlying CRC and PDAC and converged in a top‐scoring network. As expected, Network 1 included genes involved in *Wnt* signaling, epithelial–mesenchymal transition commonly promoting invasion and cell migration and other key pro‐growth pathways, consistent with the loss of the fine‐tuned KRAS molecular switch. To assess our in silico findings, we used cancer cell lines that recapitulate CRC and PDAC primary tumors. We used model systems with distinct KRAS statuses, such as pancreatic BxPC‐3 (*KRAS‐*WT), PANC‐1 (*KRAS* p.G12D) AsPC‐1 (*KRAS* p.G12D), and COLO 357 (*KRAS* p.G12D) cell lines and colorectal Caco2 (*KRAS*wt), HCT116 (*KRAS* p.G13D), SW480 (*KRAS* p.G12V), and Ls174T (*KRAS* p.G12D) cell lines. The mRNA levels of 11 network‐based genes were measured via qRT–PCR. To compare the changes in expression between our study and our in silico analysis, we reported the relative expression of mutant *KRAS* compared with that of WT KRAS in CRC and PDAC cell lines. The TCGA tissue findings revealed upregulation of the 11 network‐based genes in patients with *KRAS‐*WT tumors compared with those with WT tumors.

Our experimental data confirmed the expected changes in the expression of most genes in the CRC‐MT and ‐*KRAS*‐WT cancer cell lines (Table 1). However, compared with *KRAS*‐WT PDAC, PDAC with mutant KRAS exhibited a distinct trend in expression associated with each mutant form of *KRAS*. The common genes that exhibited concordant expression trends in CRC and PDAC *KRAS*‐mutant cells compared with their corresponding *KRAS*‐WT cancer cell lines were *MUC1*, *ITGA3*, and *PHLDA*. However, the expression of *DKK1*, a secreted inhibitor of *WNT* signaling, significantly changed in both cancer cell lines but exhibited opposite trends in CRC and PDAC cell lines. Therefore, *DKK1* had a tissue‐dependent trend‐ and context‐dependent patterns as we found distinct expression levels associated with each mutant form of KRAS in PDAC (downregulated compared with WT PDAC *KRAS* COLO‐357, *p* < 0.05). These findings are not fully surprising since context‐specific KRAS–effector (sub)complexes have been found in Caco‐2 cancer cell lines [39]. However, FSs prioritize molecules consistent with the cellular plasticity traditionally associated with KRAS‐driven cancers. MUC1 is a transmembrane protein recognized to be aberrantly expressed in a variety of tumors, including pancreatic and colon cancer [41, 42, 43, 44], and its expression is mostly associated with tumor progression and poor outcome. To date, MUC1 has shown great potential as a diagnostic marker and tumor treatment in NSCLC with KRAS mutation [45] but its role in PDAC and CRC is almost unknown. Recently, MUC1 was shown to promote PDL1 expression on tumor colon cells through the recruitment of inflammatory cytokines and subsequently inhibit the antitumor immune response via the *PDL1/PD1* signaling pathway, providing a foundation for the application of *PDL1* inhibitors in *MUC1*‐positive colon cancer [46]. ITGA3 is a cell surface adhesion protein that interacts with extracellular matrix proteins and is involved in cancer metastasis. It has been proposed to be a diagnostic and prognostic biomarker for pancreatic cancer [47, 48]. In colon cancer, *ITGA3* seems to be generated reactive oxygen species, as a byproduct of integrin engagement, where the subsequent change in redox signaling may attenuate or even cause cellular migration [49]. Recently, Duan et al. highlighted the clinicopathological characteristics, prognostic value, immune features, and functional mechanisms of PHLDA family members pancreatic adenocarcinoma. Mechanistically, these proteins are activated in multiple oncogenic pathways, and their overexpression is significantly correlated with sensitivity to multiple traditional chemotherapeutic drugs and novel targeted MEK1/2 inhibitors [50].

## 5 Conclusion

In conclusion, our study explored the challenging landscape of the *KRAS* gene, emphasizing its intricate network and molecular heterogeneity across colorectal cancer (CRC) and pancreatic ductal adenocarcinoma (PDAC) patients. Focusing on the pivotal G12D and G12V mutations prevalent in CRC and PDAC, respectively, our investigation delves into the panoramic trends of cancer‐related genes associated with the *KRAS* mutational status in these distinct cancers. Employing an integrated analysis, we harnessed machine learning‐based feature selection to identify signatures of genes converging into a top‐scoring network. Our exploration culminated in the identification of common genes—*MUC1*, *ITGA3*, and *PHLDA*—with concordant expression trends in both CRC and PDAC *KRAS* mutants. Particularly MUC1 is promising, and it seems related exclusively with KRAS mutation independently of the type of mutation. However, *DKK1*, a *WNT* signaling inhibitor, exhibited a tissue‐dependent trend and context‐dependent pattern, revealing the multifaceted interplay between *KRAS* mutations and gene expression in CRC and PDAC. Altogether, these findings, also confirmed by validation performed on public databases of cellular lines, widen our understanding of *KRAS*‐mutant driven oncogenesis shedding light on shared transcriptional signatures. Our analysis offers insights into the intricate molecular network underlying KRAS‐driven oncogenesis in CRC and PDAC, by identifying common genes such as MUC1 and unraveling their distinct expression patterns. Validation of our results on public databases adds reliability to our findings, creating the basis for future research efforts aimed at translating these findings into clinical applications.

Nevertheless, some limitations should be taken into account. First, FS methods may struggle to efficiently navigate this complexity, leading to the risk of discarding relevant biological information or, conversely, retaining noise. Moreover, FS methods often operate based on the assumption that individual genes act independently, neglecting the intricate network of interactions that characterize biological systems. Second, although we selected cancer cell lines that recapitulate the two most common CRC and PDAC subtypes, *KRAS* G12D and G12V, these cancer cells could partially explain the spectra of *KRAS*‐activating mutations and their combination with other driver genes present in individual tumors. Notably, the TCGA cohort highlighted the relevance of tumor purity in identifying to PDAC transcriptional subtypes. They found prognostic signatures based on clinical proteomic subtypes significance [51]. Recently, Li, Rajapakshe, and Maitra [48] found novel genes signatures in different pancreatic cancer subtypes. However, lately reviewed by according to Connor and Gallinger [52], there is still a need for a better consensus among the current distinct expression‐based classification systems for PDAC transcriptional subtypes. Soon, in silico approaches coupled with 3D cell culture methods known as organoids [53] deep could provide biological insights and help researchers better assess common cancer signatures even in poorly represented subtypes to better reflect the tumor individual phenotype and develop more personalized interventions. Finally, the current study focuses on mutant KRAS‐independent CRC and PDAC tumors, revealing elevated levels of MUC1, ITGA3, and PHLDA. Our findings suggest that targeting these proteins could be a viable therapeutic strategy for at least some mutant KRAS PDAC and CRC. Further research is needed to determine the relevance of MUC1, ITGA3, and PHLDA in mutant KRAS‐dependent PDAC and CRC cells.

## Author Contributions

**Katia Pane:** conceptualization (equal), data curation (equal), formal analysis (equal), methodology (equal), project administration (equal), resources (equal), validation (equal), visualization (equal), writing – original draft (equal). **Mario Zanfardino:** conceptualization (equal), data curation (equal), formal analysis (equal), software (equal), validation (equal), writing – original draft (equal). **Anna Maria Grimaldi:** formal analysis (equal), investigation (equal), methodology (equal), validation (equal), writing – original draft (equal). **Ilaria Leone:** formal analysis (equal), investigation (equal), methodology (equal), writing – original draft (equal). **Silvia Nuzzo:** conceptualization (equal), investigation (equal), supervision (equal), writing – original draft (equal), writing – review and editing (equal). **Marco Salvatore:** supervision (equal). **Monica Franzese:** conceptualization (equal), investigation (equal), project administration (equal), writing – original draft (equal).

## Ethics Statement

The manuscript does not contain clinical studies or patient data. All the data were obtained from public databases.

## Consent

All the authors read and approved the final manuscript for publication.

## Conflicts of Interest

The authors declare no conflicts of interest.

## Supporting information


**Figure S1.** KRAS protein changes available in public dataset for colon and pancreatic cancer. (A) Overlapping KRAS protein changes in primary tumors for TCGA‐COAD (colon) and TCGA‐PAAD (pancreas) cancers, respectively. (B) Unique and overlapping KRAS protein changes available in the CCLE for colorectal (large intestine) and pancreatic cell lines.

**Table S1.** qRT‐PCR specific primers.
**Table S2.** Features selection accuracy.
**Table S3.** g:Profiler enrichment analysis.
**Table S4.** Ingenuity Pathway Analysis top scoring network 1 gene annotations.
**Table S5.** Genetic features of colon and pancreatic cancer cell lines.

## Data Availability

The datasets analyzed during the current study are publicly available in the TCGA repository (https://portal.gdc.cancer.gov/).
