# Supplementary figures and images for: Feature Selection and Network‐Driven Analyses to Unveil Common RNA Signatures in Colon and Pancreatic KRAS‐Mutant Cancers
Source: Cancer Med. 2025 Feb 27;14(5):e70468. doi: 10.1002/cam4.70468 (PMC11865888; doi:10.1002/cam4.70468)

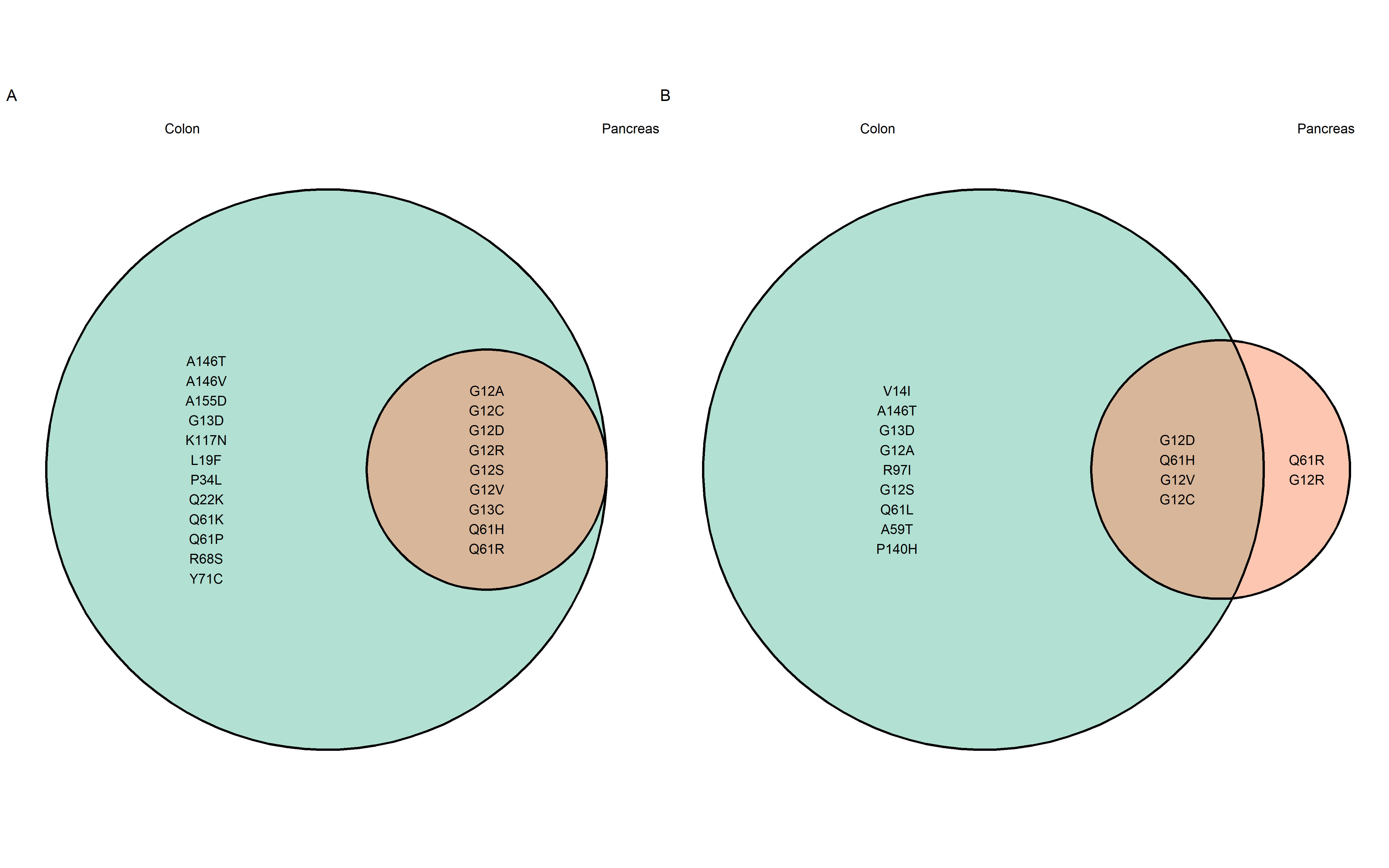

Supplement: Supplementary file 1 — Figure S1. KRAS protein changes available in public dataset for colon and pancreatic cancer. (A) Overlapping KRAS protein changes in primary tumors for TCGA‐COAD (colon) and TCGA‐PAAD (pancreas) cancers, respectively. (B) Unique and overlapping KRAS protein changes available in the CCLE for colorectal (large intestine) and pancreatic cell lines. [file CAM4-14-e70468-s001.tiff]
